# Leveraging incentives to increase HIV testing uptake among men: qualitative insights from rural Uganda

**DOI:** 10.1186/s12889-019-8073-6

**Published:** 2019-12-30

**Authors:** Alex Ndyabakira, Monica Getahun, Ambrose Byamukama, Devy Emperador, Stella Kabageni, Kara Marson, Dalsone Kwarisiima, Gabriel Chamie, Harsha Thirumurthy, Diane Havlir, Moses R. Kamya, Carol S. Camlin

**Affiliations:** 1 Infectious Diseases Research Collaboration, Kampala, Uganda; 2 Department of Obstetrics, Gynecology & Reproductive Sciences, University of California San Francisco, San Francisco, CA USA; 3 Department of Medicine, Division of HIV, Infectious Diseases, and Global Medicine, University of California San Francisco, San Francisco, CA USA; 4 Division of Health Policy, Perelman School of Medicine, University of Pennsylvania, Philadelphia, PA USA; 5 Center for Health Incentives and Behavioral Economics, University of Pennsylvania, Philadelphia, PA USA; 6 School of Medicine, College of Health Sciences, Makerere University, Kampala, Uganda

**Keywords:** HIV testing, Men, Economic incentives, Lottery, Loss aversion, Sub Saharan Africa

## Abstract

**Background:**

Few studies have explored how economic incentives influence behavioral outcomes. This study aimed to identify pathways of action of an incentives-based intervention to increase men’s participation in HIV testing.

**Methods:**

The qualitative study was embedded in a randomized-controlled trial that compared effectiveness of gain-framed, loss-framed and lottery-based incentives to increase HIV testing among men. Following testing at a community health campaign, 60 in-depth interviews were conducted with men systematically sampled on the basis of age, incentive group, and campaign attendance. Data were coded deductively and inductively for thematic content analysis.

**Results:**

Incentives addressed men’s structural, interpersonal and individual-level barriers to testing: offered at convenient locations, incentives offset costs of testing, in lost wages, which are exacerbated when livelihoods required mobility. Interpersonal barriers included anticipated stigma/fear of disclosure, social obligations, and negative peer influences. Providing incentives in public settings provided “social proof” that prizes could be won, and facilitated social support and positive norms by promoting testing with trusted others. Incentives had little influence when men appraised prize values to be low, disbelieved they would win a prize, or were already intrinsically motivated to test. Yet, incentives provided a behavioral ‘cue to action’ for many men who perceived themselves to be susceptible to HIV and perceived HIV disease to be severe, acting as secondary motivator for testing that “*sweetened the deal*”.

**Conclusion:**

Incentives can be an important ‘lever’ to promote men’s healthy behaviors in resource-poor settings. HIV testing in convenient, public settings, when paired with incentives, provides multiple pathways to stimulate men’s testing uptake.

**Trial registration:**

Registered with ClinicalTrials.gov on 08/10/2016, ID: NCT02890459. The first participant was enrolled on 11th April 2016.

## Introduction

Across sub-Saharan Africa, men and boys living with HIV are 20% less likely than women and girls living with HIV to know their HIV status, and 27% less likely to be accessing treatment [1]. Globally, men test for HIV at lower rates even in the context of innovative community and home-based HIV testing approaches [2]. Economic incentives have been shown to promote a number of health behaviors in both high- and low-income countries [3–5] and to increase HIV testing at workplaces in South Africa [6], at community level in rural Uganda [7], and among Zimbabwean couples [8]. However, limited data exist to explain why incentives work, or fail, if they do; a deeper understanding of the pathways of action is needed to facilitate the widespread integration of incentives in public health interventions.

We conducted a six-arm HIV testing incentives randomized trial and compared the effectiveness of different novel incentive interventions to increase HIV testing among men [7]. The trial drew upon observations from the field of behavioral economics that gain-framed incentives (providing a small reward for carrying out a behavior) can act upon individuals’ tendency to delay behaviors for which costs may be immediate whereas benefits may lie in the future; that offering lotteries with low probabilities of winning large prizes may be more appealing than gain-framed incentives, at equivalent per-person programmatic cost, because people generally pay greater attention to the magnitude of a reward than the probability of winning; and finally that people display loss aversion in their decision-making: gaining something of value is less motivating than losing something of equal value. The study compared the effectiveness of three incentive types: standard fixed (i.e. gain-framed) incentives (control); loss-framed incentives; and lottery-based incentives. Each incentive type had a low and high amount, with an expected value of about US$1 and US$5 per participant, respectively. The low value gain- and loss- framed incentives included similar non-monetary gifts such as a wash basin, a bar of washing soap, scratch airtime and packets of salt, while high value incentives included gumboots, branded T-shirts, a hand hoe, and a panga. The low value incentives for the lottery arm included radios and telephone handsets while high value items included bicycles. We found that during a 13-day community health campaign (CHC) that offered HIV testing and counseling along with multi-disease screening services, 75 to 80% of men enrolled in the trial attended and tested for HIV—higher participation than previously described at other, similar campaigns in the region [7]. Men enrolled in the low-cost lottery arms tested for HIV at a higher proportion than men in the other incentive arms. In this qualitative study embedded within the trial (with analyses conducted in parallel with the quantitative analyses referenced above), we explored men’s perceptions, attitudes and preferences related to incentives, men’s attributions for their decision-making related to testing, including the relative influence of incentives, as well as men’s other motivations and barriers related to HIV testing. We sought to compare emergent themes across groupings of men by whether or not they tested for HIV at the campaigns, and to ascertain any variations by incentive types.

## Methods

### Study design

Data are from a qualitative study embedded in the ‘Innovative Incentive Strategies for Sustainable HIV Testing and Antiretroviral Treatment’ trial (NCT02890459); study details are published elsewhere [9]. Briefly, we conducted a full household census in one community in rural Southwestern Uganda, and randomized into the trial all eligible men who consented to participate. All community members were informed about the CHC dates and venues through community announcements, and invited to come for multi-disease screening. The 13 CHC days were evenly distributed across the study community and the CHC changed location every day. For sites that were considered high volume, testing occurred at same location for two consecutive days. All community members were free to attend CHC and have disease evaluation from any location of their choice. After the CHC was completed, we conducted qualitative research to examine the barriers and motivators for HIV testing among men, with a focus on perceptions of different incentive strategies and their influence on HIV testing.

For the qualitative study, following the 13-day campaign, 60 men were purposively selected from groupings based on their age, incentive group, and whether or not they attended the CHC and tested for HIV. In-depth semi-structured interviews (IDIs) were conducted using guides developed for this study (attached as Additional file 1) with a mix of participants from the different study arms and HIV testing groups, further stratified to obtain a mix of participants from 18 to 24, 25–44 and 45 and older age groups (Table 1).

**Table 1:** Qualitative sample

Characteristic	Low amount			High amount			Total
	Fixed	Loss aversion	Lottery	Fixed	Loss aversion	Lottery	
N	10	10	10	10	10	10	60
Age [n (%)]							
18–24	3 (30)	5 (50)	4 (40)	4 (40)	7 (70)	3 (30)	26 (43)
25–44	5 (50)	3 (30)	2 (20)	4 (40)	2 (20)	5 (50)	21 (35)
45+	2 (20)	2 (20)	4 (40)	2 (20)	1 (10)	2 (20)	13 (27)
Parish [n (%)]							
Mabira	5 (50)	2 (20)	5 (50)	2 (20)	3 (30)	1 (10)	18 (30)
Katyazo	4 (40)	4 (40)	3 (30)	5 (50)	7 (70)	5 (50)	28 (47)
Ruhunga	1 (10)	3 (30)	1 (10)	3 (30)	0 (0)	2 (20)	10 (17)
Itara	0 (0)	1 (10)	1 (10)	0 (0)	0 (0)	2 (20)	4 (7)
HTC [n (%)]	5 (50)	5 (50)	5 (50)	5 (50)	5 (50)	5 (50)	30 (50)

### Data collection and analysis

A team of three trained qualitative researchers conducted in-depth semi-structured interviews in the local language, Runyankole, lasting 45 to 60 min. The in-depth semi-structured interview guide explored topics informed by both the behavioral economics concepts described above as well as theories of health behavior that are salient for exploring HIV testing decision-making [10–12]. The topics included men’s feelings about and appraisals of the incentives, their stated motivations and attributions for their decision whether or not to test, including barriers and the relative influence of incentives on that decision, and social and relational influences (peers, partners, and family) on the decision to test. Audio recordings of IDIs were translated, transcribed into English, and deductively and inductively coded, drawing upon constructivist grounded theoretical approaches [13]. An initial coding framework was developed on the basis of the theory-informed domains of inquiry for interview guides; these a priori codes were applied using qualitative analysis software by a four-person team under the guidance of the lead investigator. The coding framework was iteratively refined during data collection and analysis, following review and discussion of empirical findings. Coded excerpts were extracted, reviewed and analyzed for further reductions into the analytical themes presented below. All quoted excerpts presented include a brief description of the participant’s age, whether or not he participated in HIV testing and counseling (HTC vs. non-HTC, respectively), and to which of the six study groups the participant was randomized (fixed [gain-framed], loss-framed, or lottery-based incentives, at either low or high incentive amounts).

### Ethical approvals

All participants provided informed consent to participate. The study was approved by the institutional review boards of Makerere University *(SOMREC 2015–138)*, the Uganda National Council for Science and Technology *(SS3980)*, the University of California, San Francisco *(15–16,876)*, and the University of Pennsylvania.

## Results

Men discussed their barriers and motivations related to CHC attendance and HIV testing uptake. We drew upon the social ecological model for interpreting ways these barriers and motivations for men’s testing acted at structural, interpersonal, and individual level domains of influence, and to deepen our understanding of the role of incentives in influencing HIV testing uptake. Below are excerpts that highlight men’s navigation of HIV testing and the role of incentives in navigating testing decisions.

### Structural barriers and motivators to testing, and the role of incentives

*Influence of livelihoods and mobility on HIV-testing uptake, and effect of CHC testing location/proximity to community on these livelihood-related barriers.* Men discussed having foregone HIV testing in the past, because taking the time to go to a clinic to test for HIV conflicted with their work or income-generating activities. A man described how, in the past, he lacked funds to travel to a health facility to test and often missed meals during the long wait times at the clinic:

*“Every other time, I had to get money for transport to go to [the nearest health center] where I would at times spend a day without a meal. Now the service was brought closer in addition, gifts [incentives] were to be given. I would not miss the chance to attend [...]” (25–44 year-old, participated in HIV testing and counseling [HTC], fixed, low).*

A man discusses having had to prioritize time to search for work over HIV testing; the health campaign helped him find time to test as the services were brought closer:

*“I had spent a long time without testing because am always busy looking for money so one would not get time even to go to [nearest health center] get tested. So when I got the chance to get tested in addition to getting a gift* [incentive]*, I said it was a must to attend, I also called the other workers so we went to get tested.” (25–44, HTC, fixed, low).*

The convenient location of the CHC within the community, and the ability to minimize or avoid transportation costs motivated many to attend the CHC. In the following quotes, participants discuss the location of the CHCs as a motivator to participate:

*“The gift did motivate me but mostly was that services were brought nearer to us and the gift made my decision easier to go test.” (44+, HTC, fixed, low).*

*“[...] Since I was using money for transport to get tested together with her [his wife], I was not going to miss a chance of testing for free nearby. So that is why we decided to get tested.” (25–44, HTC, fixed, high).*

*Influence of livelihoods and mobility on HIV-testing uptake, and effect of incentives on these livelihood-related barriers.* Men in the setting often engaged in informal sector livelihoods that required them to travel away from the community; both long work hours and time spent traveling to and from the community presented a challenge to attending CHCs during the hours they were running. Men weighed the costs of lost income if they attended CHC against the benefits of testing, the benefits of the incentives, or both. Narratives revealed that incentives helped overcome livelihood-related barriers for some men via offsetting the perceived costs of testing; for others, and especially men with steady employment, it was not a motivating factor. Men considered the value of incentives prior to participating in the CHC. For example, some men in lottery groups perceived their chances of winning to be low, and opted for alternative activities that were perceived to guarantee some income. These decisions to choose livelihood opportunities over HIV testing were overwhelmingly voiced by younger, working, and highly mobile men as illustrated in the quotes below:

*“There was nothing much [to lose] since these were not expensive prizes that it would hurt a lot if you missed. The prizes were of a low value.”(25–44, non-HTC, fixed, low).*

*“I had won a bicycle but I got busy and never tested. A bicycle looked like a small prize, I thought there would be cars, cows and other expensive prizes but the prizes were of low-value. Since I had a lot of work to do and I would get more money, I went with the better deal.” (25–44, non-HTC, lottery, high).*

Yet even among older participants in fixed and loss-aversion groups, who were essentially guaranteed a prize, some expressed reticence about testing, and shared that the perceived loss of income while attending the CHC discouraged their attendance.

### Interpersonal barriers and motivators to testing, and effect of incentives on these barriers

Interpersonal level barriers to men’s testing included anticipated stigma and fears of disclosure, social obligations, and negative social/peer influences in which testing is not normative. Below, men discuss how anticipated stigma and fear of disclosure – particularly to their wives - inhibits men from participating in testing:

*“When women know that we [men] are HIV-positive, problems start. Even mine [wife] tells me that if am found sick, she can kill me or separate from me. I also ask if it were them [women] sick, not me, what would happen? That is why men fear. Rather stay without knowing.” (25–44, HTC, fixed, high).*

*“Some [men] fear telling their wives of positive results. That is why they want their wives to go test first. Or others, after testing and they’re positive, they hide the results from the wife and start taking medication from somewhere else but not home [closest health center].”(25–44, HTC, fixed, low).*

Anticipated stigma from others in the community also proved to be a significant barrier to HIV-testing:

*“If one goes to a health facility, there are many kinds of people— even those from your village— and they may spread rumors that so-and-so is HIV-positive, even when you may not be sick.” (25–44, non-HTC, loss aversion, high).*

Yet other narratives revealed that HIV testing was normative among some men, in the context of relationships in which men felt supported by their partners, or tested together with their partners. In particular, men with peer and intimate partner support discussed the influence of partners and peers on their motivations to test, and their prior participation with others in HIV testing and health-related activities. In the excerpts below, men discuss their histories of couples’ HIV-testing:

*“My wife and I decided that we would test for HIV and get the results.”(25–44, HTC, lottery, low*).

*“When you and your wife decide, you go because if you don’t your wife may start questioning why you never go to test. When one goes with the wife for the checkup, you test together and she doesn’t ask many questions.”(45+, HCT, loss aversion, low).*

*“She [my wife] said that why are you afraid of testing and she encouraged me to go ahead and get tested. My wife is the one who supported me to test*.” *(45+, HTC, fixed, high).*

Below, men discuss the role of peer influence in their decision to take up HIV testing:

*“One goes to know his/her health status and my other friends were going too, so I got the motivation to go and test also.” (18–24, HTC, loss aversion, high).*

*Peer-related influence of incentives:* Providing incentives in public settings enabled many to view evidence that prizes could be won, a form of what is termed “social proof” in the field of social marketing. Witnessing other men who had won incentives that were perceived to be of high quality and value was also helpful in overcoming barriers that would have otherwise prevented men from testing.

*“There is someone that won a bicycle and a phone, so they [the incentives] encouraged me to go for testing.” (25–44, HTC, fixed, low).*

*“When a person came with a gift [won an incentive], friends who had no time created [time] to go get tested; thus, I saw that the gift motivated many people to go get tested, but if it wasn’t for the gift, you know us men, we wouldn’t get time to go get tested.”(25–44, HTC, fixed, high).*

*When incentives and other motivations to test ‘didn’t work’: men’s social obligations.* Despite their interest, some men reported that they were unable to attend the CHC and test for HIV due to unforeseen social obligations, including attending funerals, childbirths, or care-giving for others. Below, men describe how funerals and other obligations prevented them from attending the CHC:

*“I could not leave people who had come for burial [at my home] and go to the campaign. I later heard you had gone to Runengo [CHC site] but there were other people coming at home. I hoped if it [CHC] came next time I would attend.” (44+, non-HTC, lottery, low).*

*“He [study team member] told me to come and test but unfortunately on the day of testing, we lost someone and I went to bury thus could not attend.” (25–44, non-HTC, lottery, low).*

### Individual level barriers and motivators to testing, and the role of incentives

*Individual-level barriers: mistrust and low perceived value of incentives.* Individual level barriers to HIV testing uptake included negative perceptions of the incentives as well as mistrust of the motives of researchers. Some men perceived the prizes to be of low value:

*“There should be an increase in the value of prizes [incentives] so that we know they can be used to earn, and in that we are motivated to attend the campaign” (25–44, non-HTC, lottery, high).*

Some men also expressed skepticism about the prizes and doubt that the incentives were real. Below, men describe how they were surprised to learn that incentives were real:

*“I thought it was a lie [incentives], but I progressed and took the test, and when the doctor tested me, he told me to move somewhere and I received my gift and went home very happy”(25–44, HTC, loss aversion, low).*

“*I thought they were lying and just wanted people to get tested, but later it turned out they were telling the truth”(18–24, non-HTC, lottery, high).*

Men describe how they never bothered to discuss incentives after the study team had left, because they never believed that the incentives were real:

“*We did not talk much about the gifts because we doubted their availability and thus waited to speculate because once, we participated in some things way back, and they were not successful.” (18–24, non-HTC, lottery, high).*

A man explains how he could not imagine that an organization can offer health services and in addition give free incentives, and was surprised when this happened:

“*I had no questions but thought that these people just wanted us to come and test, but I doubted the presence of the prizes. I could not imagine giving all people prizes because it would be expensive, but it happened and I know some who got big prizes like bicycles.”(18–24, non-HTC, lottery, high).*

Other men did not attend the CHC because they perceived they had a low probability of winning a prize, as illustrated in the following quote:

“*But also there were few bicycles to be won in the whole sub-county and so my chances of winning were less. I had some work to do and I was already late, and I knew my chances were less, so I was not motivated” (18-24, non-HTC, lottery, low)*

*Other individual-level barriers to testing.* Some men didn’t view CHC attendance and HIV testing as particularly urgent because they had previously tested for HIV, or they preferred the privacy of clinic-based testing. Therefore, they decided not to attend, as illustrated in the following quotes.

*“I also knew your mission was to test those men that do not like testing, but as for me, I have tested. And no need to go there for me, to go public, and I am forced to show people even when I do not want to tell you. That is why I go to Bwizibwera Health Centre where it is private” (18-24, non-HTC, lottery, high)*

Other individual-level barriers included more interest/concern about other diseases, low perceived severity of HIV, and fatalism related to HIV. Below, a man described being more interested in screening for other diseases than HIV, suggesting that though screening for other diseases (including hypertension and diabetes) was offered at the campaign, not all participants reported awareness of the multi-disease services available:

*“Those that had come from testing were telling me it was just HIV testing and to me that was not very important. I was more interested in testing for diabetes, pressure [hypertension], hepatitis B. Hepatitis is now common and it kills so I was much more interested in that.” (25-44, non-HTC, lottery, high)*

Perceptions of HIV as either a low-severity illness or a fatal condition, were both expressed as barriers to testing for HIV. Some young men expressed a perception of HIV as a low-severity illness due to the availability of efficacious antiretroviral treatment (ART). ART was perceived to improve health and help maintain daily routines, in the event of an HIV-positive diagnosis. A man describes how HIV is no longer seen to be a life-threatening condition since there is effective medication:

“*HIV is taken to be less frightening nowadays and a person can start medication when they feel like, and live […]” (18-24, non-HTC, lottery, high)*

*“Others say that they no longer fear, since there is medication*” (25-44, non-HTC, lottery, low)

Yet, other men were reluctant to take up testing out of fear of dying after being diagnosed as HIV-positive:

*“Some [men] get involved while others fear because they think if I am found positive, I would die quickly. so they better go when they are already bedridden.” (25-44, non-HTC, fixed, low)*

*Intrinsic motivations to test for HIV.* Men also described their intrinsic motivations to test for HIV, and their positive attitudes towards health services in general. In particular, older men and men in local leadership roles discussed intrinsic motivations to test and their prior participation in HIV testing and other health-related activities. In these narratives, participants share that the incentives did not have a major role in influencing their decision to retest, further stating their desire to attend even in the absence of incentives, as shown in the following quotes.

*“I did not see the incentives as very important because I am aware that knowing my health status was more important.” (25-44, non-HTC, fixed, high)*

*“I was not much interested in the prizes but my health. I was negative which I was happy about though I never got a prize.” (45+, HTC, lottery, high)*

Men expressed a desire to attend and confirm the veracity of the CHC and services offered, as well as the accuracy of the HIV tests, and to confirm their HIV status.

*“I came to see how this campaign operates, is conducted, whether good or bad and rate it; but I was very grateful for the way you operated entirely.” (25-44, HTC, lottery, low)*

*“I knew that I was positive and so I wanted to come and check and see if your machines do work.” (25-44, HTC, fixed, low)*

*Among intrinsically-motivated men: incentives as “cue to action”.* Among men who were intrinsically motivated to test, many described incentives as a secondary motivator for HIV testing. They discussed prior participation in health campaigns or efforts, and a general willingness to test. These men were already participating in other health efforts within the community, prior to the arrival of the CHCs, but were prompted to participate because of the incentives. A man discussed his prior testing experience and described the incentives as a secondary motivator that “*sweetened the deal*”:

*“Now you are asking me how the gift motivated me to go for checkup. As for me, I had always gone for checkups, even that gift added on motivating me to check because I had spent time without going to the health center to know my health status. Getting HIV testing close by motivated me.” (25-44, HTC, fixed, high)*

### Emotional responses to incentives

Men across incentive groups and age groups described general happiness about the prizes, and discussed at length their happiness on winning a prize, overshadowing discussions about concerns they had had about testing. Below, a man describes the happiness he experienced on winning and receiving the prizes:

*“I felt very happy since I and my wife had both tested HIV negative and I had received the actual gift I had won. It felt like I had gone to work and returned with salary.” (45+, HTC, loss aversion, low)*

*“When I got the gift, I was happy because there are others that did not get any and my testing went well, I was motivated by the gift to test and get it. I did not want to miss because they would take it back.” (18-24, HTC, loss aversion, high)*

Other participants described positive perception of incentives depending on incentive group: feelings and language of ownership were expressed among men in the loss-framed incentive groups, while men in the lottery groups expressed excitement upon winning a prize*.*

Participants in the loss aversion group express the language of ownership:

*“When the doctor tested me, he told me to move somewhere and I received my gift and went home very happy” (25-44, HTC, loss aversion, low).*

*“ I knew I would get my hoe and I had to come from where I was to receive it, because I am not usually at home” (25-44, HTC, loss aversion, high)*

A man in the lottery groups expresses the excitement he got on winning the reward:

*“[…..] after being tested and given a gift I got happy, too much excitement” (44+, HTC, lottery, low)*

Some described the usefulness of incentives in overcoming livelihood challenges and improving life. To these the usefulness rather than the value of the incentive was likely the more motivating factor. A man describes how the entire family utilizes the gum boots [incentive] whenever it rains:

*“I feel proud. Now days it rains so am always putting on my boots while gardening. At times my wife and children put on the boots. I was very pleased with the gift.” (25-44, HTC, fixed, high)*

A man describes how his main focus in testing for HIV was the anticipated utilization of the incentive:

*“I had to be there to test and get my prize so my hoe would help me to dig. 'It looked good to me, I got my hoe because it does many things for me. I felt happy.” (25-44, HTC, loss aversion, high)*

Some men discussed the morale-boosting effect of the incentives, including men who did not attend the campaign but described witnessing the morale-boosting, and trust-building effect among other community members.

*“Your gifts were powerful. They gave people morale […] I had two reasons [for attending]… I wanted to know my HIV status and also pick my gift since I had already won.” (25-44, HTC, fixed, low)*

*“Prizes entice people to get involved in government campaigns. Ugandans like being motivated. People would have been few at the campaign if prizes were not being given. If one wins boots, bicycle or radio, they go away happy.” (25-44, non-HTC, lottery, high)*

## Discussion

This qualitative study, embedded within a clinical trial testing the effectiveness of incentive type and amount on men’s HIV testing uptake, revealed pathways through which incentives appear to influence men’s uptake of testing, as well as the potential limits to incentives-based interventions. The findings can help to inform an understanding of the contexts and circumstances in which incentives may or may not be effective at influencing behavior change in resource-poor settings. How did incentives ‘work’ to increase participation in HIV testing among rural Ugandan men? **At the structural level**, incentives worked as intended for many men to offset the costs of taking time away from work to participate in testing (costs that are exacerbated when men’s livelihoods required mobility— travel away from home communities where testing campaigns took place). Men’s work-related barriers to care-seeking (including participation in HIV testing) are underpinned by gender norms that reinforce men’s identities as workers, earners, and providers for families and households [14, 15]. Fulfilling this male gender role expectation is of utmost importance for men and can be quite difficult in high poverty settings; thus, activities that might detract from income-earning have lower priority unless their potential value offsets perceived costs. Findings showed that many men carefully weighed costs and benefits of testing, with the perceived value of incentives factoring in to their decision-making. In addition, *how* the incentive intervention was delivered mattered: incentives for testing were offered at health campaigns that were conveniently located near home and work settings. This further worked to offset costs of time taken away from work, as well as perceived inconvenience relative to traditional clinic-based approaches to testing for HIV.

The public nature of the health campaigns– another factor related to *how* the intervention was implemented– also facilitated **interpersonal-level influences** on men’s motivation to test. The testing campaigns, held in open settings with a festive atmosphere and with multi-disease screening included, provided opportunities for demonstrations of social support among men, for the circulation of new positive norms related to testing for men, and vicarious efficacy [16] for HIV testing, via their experience of participating in testing campaigns with friends. Further, situating the incentives intervention in a public arena appears to have amplified its power for many men via presenting a ‘social proof’ of the possibility of winning, as men viewed other men leaving the campaign with prizes in hand. On the other hand, interpersonal, peer influences also could be negative, we found, when friends did not attend campaigns, or expressed negative attitudes or beliefs about incentives or about HIV testing to their peers.

The influence of incentives on men’s participation in testing was not operant under certain conditions: when men appraised the value of incentives to be low, the perceived time and income costs of participating in testing weighed more highly against taking the time to test at the campaign. Similarly, when men simply didn’t believe that they were likely to win a prize (or didn’t believe the prizes were real), incentives had no effect on their decision-making. We found that in many instances incentives would have influenced men to test, but obligations— a funeral, a work event, a family emergency— simply interfered. Finally, the incentives did not always work to facilitate some men’s ability to overcome their expressed anticipated stigma and fears of disclosure of an HIV-positive status. It was beyond the scope of the trial to include specifically an intervention element targeted to women, couples, or female partners of the men in the communities, in order to directly address the concerns related to disclosure with partners.

The findings also suggest a set of **psychological pathways** through which incentives influenced men’s testing. As hypothesized (following precepts of theories of health behavior) the incentives appeared to provide a behavioral ‘cue to action’ [17] for men who perceived themselves to be susceptible to HIV and who perceived HIV disease to be severe. Moreover, for men who appraised the severity of HIV infection to be low (e.g. ‘these days, malaria is a bigger problem than HIV’), their perceived risk of HIV would not motivate a desire to test— and providing an incentive (and possibly multi-disease services, as well) added a necessary motivator to participate in testing.

In numerous accounts, men described their positive emotions about participating in the campaigns because of the prizes, including excitement, happiness, and a preoccupation with thinking about the prizes in advance of the campaign event, and an anticipated sense of ‘ownership’ - particularly in the loss-framed group (an intended perception, as the investigators sought to generate a sense of loss aversion in the loss-framed intervention group). These emotions evoked by the incentive interventions may have functioned to distract men from negative emotions related to testing that they may have held (i.e. fears), and may also have facilitated positive emotional associations with testing. Circumstances in which this pathway was not operant— from narratives without accounts of these positive emotions — included those in which men appraised value of incentives to be low, when men felt mistrust towards the health system or study team, when men felt they were unlikely to win a prize, and when men were already intrinsically motivated to test for HIV for reasons entirely unrelated to incentives.

Our study was subject to limitations. Principally, although we had hoped to ascertain differences in the effects of different incentive intervention modalities tested in the study, as well as in the value levels of incentive types, emergent findings did not reveal distinct patterns and themes across the study’s intervention groupings. It is possible that a larger qualitative sample would have permitted more complete data saturation, but this was not feasible within the study schedule and budget. Despite this, the findings yield important insights that can inform future programmatic efforts to elicit men’s greater involvement in health seeking behaviors such as HIV testing through the use of economic incentives.

## Conclusions

In conclusion, first, this study reinforces that incentives can be an especially important ‘lever’ to promote men’s healthy behaviors in resource-poor settings in which labor markets are volatile and men struggle to attain steady employment. The study’s findings reveal that how incentives are offered matters: pairing the public delivery of incentives with testing campaigns in community settings gives opportunities to provide proof that prizes can be won, and also can facilitate social and interpersonal support for testing by eliciting men’s engagement in testing along with trusted others (friends, partners, and families). While some men may continue to prefer home-based testing, this study suggests that campaigns in community settings, when paired with incentives, provide multiple potential mechanisms to stimulating men’s testing uptake.

Secondly, knowing the ways in which gender systems intersect with men’s labor and livelihoods, efforts to promote men’s participation in HIV testing could be amplified by messaging that underscores how knowing one’s status (and seeking prompt treatment with ARVs if needed) helps men to maintain their role as family providers and supports their ability to be successful in their work. Messaging should also address an emerging phenomenon— in the era of ARVs, with many people successfully engaged in care and displaying health and vitality— of perceived low severity of HIV disease, among young men in particular, and reinforce the reasons why HIV disease should be avoided if HIV-uninfected, and diagnosed early if HIV-infected. Future efforts to increase men’s testing should ideally also involve women or linkage to supported disclosure services for couples in order to help men who avoid testing because they fear disclosure.

## Supplementary information

**Additional file 1.** “IDI Guides Ibis 9.13.16.doc” Title: Innovative Incentive Strategies for Sustainable HIV Testing and Linkage to Care: In-Depth Semi-Structured Interview (IDI) Guides. Description: The document contains two in depth semi-structured interview guides used in the Innovative Incentive Strategies for Sustainable HIV Testing and Linkage to Care (Ibis) study.

## Data Availability

The data analysed during the current study are derived from qualitative in-depth interview transcripts. Data excerpted to protect participant confidentiality are available from the senior author on reasonable request.

## Abbreviations

ART: Antiretroviral therapy
ARVs: Antiretroviral medications
CHC: Community health campaign
HIV: Human immunodeficiency virus
HTC: HIV testing and counselling
IDIs: In-depth semistructured interviews
