# Supplementary material for: Leveraging incentives to increase HIV testing uptake among men: qualitative insights from rural Uganda
Source: BMC Public Health. 2019 Dec 30;19:1763. doi: 10.1186/s12889-019-8073-6 (PMC6937741; doi:10.1186/s12889-019-8073-6)
Supplement: Supplementary file 1 — Additional file 1. “IDI Guides Ibis 9.13.16.doc” Title: Innovative Incentive Strategies for Sustainable HIV Testing and Linkage to Care: In-Depth Semi-Structured Interview (IDI) Guides. Description: The document contains two in depth semi-structured interview guides used in the Innovative Incentive Strategies for Sustainable HIV Testing and Linkage to Care (Ibis) study. [file 12889_2019_8073_MOESM1_ESM.docx]

**Innovative Incentive Strategies for Sustainable HIV Testing and Linkage to Care**

**In-Depth Semi-Structured Interview (IDI) Guides (15 Feb. 2016; for protocol v.2.0)**

*IDI GUIDE A.1: HTC group: i) Men randomized to lottery-based incentives who attended CHC/tested; ii) Men randomized to loss aversion who attended CHC/tested; iii) Men randomized to fixed incentives who attended CHC/tested. IDI GUIDE A.1 is designed to understand: a) the pathways through which the incentives succeeded or failed; and b) the psycho-social characteristics and contexts of men who do and do not respond to incentives. Specific follow up questions that are relevant for participants in groups i) through iii) are inserted in the guide.*

*IDI GUIDE A.2: Non-HTC group: i) Men randomized to lottery-based incentives who did not attend CHC/test; ii) Men randomized to loss aversion who did not attend CHC/test; iii) Men randomized to fixed incentives who did not attend CHC/test. IDI GUIDE A.2 is designed to understand: a) the pathways through which the incentives succeeded or failed; and b) the psycho-social characteristics and contexts of men who do and do not respond to incentives. Specific follow up questions that are relevant for participants in groups i) through iii) are inserted in the guide.*

**IDI Guide A.1: With men from each study group who attended CHC & participated in HTC**

My name is ___________________. I work for the IBIS Health Project as a researcher. We would like talk to you to learn about your experiences as a man who was offered a prize, or incentive, to participate in HIV testing in this community. **We are interested in the** **needs and preferences of men in this community related to HIV testing.** We would like to learn more about how you felt about the incentive you were offered or learned about, and what other factors you considered in your decision whether or not to test for HIV.

The information that you provide will be used to inform efforts to provide programs and services for men in Uganda and other countries. This discussion will take around one hour. If you have questions you want to ask on other topics, I can try to answer them after the end of the discussion. In order not to lose any of the valuable information you tell me, I will record our conversation. After a transcript of the audio recording is typed up, the recording will be destroyed. Your name will not be attached to the recording or the transcript, and only members of the study team will be permitted to listen to what you say.

**NOTE TO INTERVIEWER: Read the consent form for in-depth interviews out loud in the participant’s preferred language and give him a copy. Ask him if he has any questions, and answer them. Ask him to sign if he agrees to participate. If the participant agreed to participate, you may start recording here, without recording any names or identifying information.**

1. ***Perceptions, attitudes and preferences related to incentives offered***
   1. I want to start by asking you about the first contact you had with our study team after the initial census visit to your household. Let’s take a minute for you to recall the day our study team member came and told you about the prizes offered to men who come to the Community Health Campaign to participate in HIV testing.
      1. Can you remember that day? Where were you? What were you doing at the time?
      2. Can you tell me, please, what did the study team member tell you about the prize?

- *[for Loss Aversion-based Incentives group:]* How did the prize look to you?
  - 1. And what was your first reaction to learning about this prize?
- And what else were you thinking, about the prize?
- How did it make you feel, to hear about the prize?
  - 1. Do you remember what else you talked about with the study team member?
- Were there any questions you asked? Were there questions you had, but didn’t ask the team member at that time?
  1. Next I want to ask you about what you did and talked about with important people in your life, about the prize and the HIV testing, after the study team member left. What happened next, that day?
     1. Who did you tell about the prize you were offered?
- Why did you choose to talk about it with that person?
- And how did they respond, when you told them?
- Were there other people you wanted to talk about it with, but felt you couldn’t? Please tell me more about that.

1. ***Motivations and barriers related to decision to participate in testing***
2. Let me ask you some more about that prize, and about your decision to come to the Community Health Campaign to participate in HIV testing. How did it affect your decision-making about whether or not to come to the campaign to get the test?
   - 1. Please tell me more about that. *Probe for why the respondent came to CHC for HIV testing.*
     2. What other issues did you consider, when you were deciding whether or not to come to the campaign for testing?
     3. Was this the first time you had ever had an HIV test?

- *[If yes*:] What worries or concerns did you have about HIV testing?
  - Did you talk with anyone about your decision to test? Why did you choose that person to talk with? How did they respond?
  - Was there anyone else you wanted to talk with about this, but felt you couldn’t? Please tell me more about that.
  - How did you feel, at the time you learned your test result?
  - And how are you feeling about it, now?
- [*If no*:] Please tell me more about your previous experience getting tested for HIV. *Probe for testing history and whether respondent has tested regularly or frequently*. What worries or concerns did you have the first time you were tested for HIV?
  - Did you talk with anyone about your decision to test? Why did you choose that person to talk with? How did they respond?
  - Was there anyone else you wanted to talk with about this, but felt you couldn’t? Please tell me more about that.
  - How did you feel, at the time you learned your test result that first time?
  - And this most recent time that you tested, how did you feel about learning your test result?
  - Was there anything different about your experience getting tested for HIV this time?
- Now I want to ask you about the time after testing, when you received the prize you were offered. How you were feeling about the prize at that point?
- Looking back, please tell me how much influence do you think that prize had, on your decision to test for HIV?
  - What were the other reasons why you decided to get tested for HIV?

1. ***Relationship, family and other contextual factors related to incentives and testing***
2. Now I want to ask you about how other men in your community feel about getting tested for HIV, especially your friends. How do you think most men feel about getting tested for HIV?
   - 1. To what extent do you and your friends talk about HIV testing? What kinds of things do your friends say about it?
     2. What are the main motivations for men to get tested for HIV?
     3. Please tell me, what do you think are the main reasons why men **do not** get tested for HIV?
     4. What other barriers do men face, related to HIV testing?
     5. What about other medical services—how do most men in this community, especially your friends, feel about accessing medical care?
3. I want to ask you more about your main intimate and family relationships, and about how you and your partners have handled discussions around testing for HIV. First, please tell me about your marital status, and about any girlfriends?

*Note: Remind respondent about Informed Consent form and human subjects protections. Interviewers should encourage respondents to report on all of their sexual partnerships, including non-marital partners. Probe for whether respondent has a wife or main partner with whom he resides, and any other sexual partners he sees regularly.*

- - 1. [If currently married] Is HIV testing something you and your wife have talked about? Please tell me about that.
- What are the things that make it hard to discuss HIV testing with her?
- What do you think might make it easier?
  - 1. [If respondent has non-marital sexual partners]: Is HIV testing something you have talked about with your girlfriend[s]? Please tell me about that.
- What are the things that make it hard to discuss HIV testing with her?
- What do you think might make it easier?

1. Is there anything else you’d like to share with me, about your experience testing for HIV, or about the prizes offered to men in this study after testing for HIV?

THANK THE RESPONDENT FOR HIS PARTICIPATION, AND COMPLETE REFERRAL PROCESS AS NEEDED.

**IDI Guide A.2: With men from each study group who did not attend CHC/participate in HTC**

My name is ___________________. I work for the IBIS Health project as a researcher. We would like talk to you to learn about your experiences as a man who was offered a prize, or incentive, to participate in HIV testing in this community. **We are interested in the** **needs and preferences of men in this community related to HIV testing.** We would like to learn more about how you felt about the incentive you were offered, and what other factors you considered in your decision whether or not to test for HIV.

The information that you provide will be used to inform efforts to provide programs and services for men in Uganda and other countries. This discussion will take around one hour. If you have questions you want to ask on other topics, I can try to answer them after the end of the discussion. In order not to lose any of the valuable information you tell me, I will record our conversation. After a transcript of the audio recording is typed up, the recording will be destroyed. Your name will not be attached to the recording or the transcript, and only members of the study team will be permitted to listen to what you say.

**NOTE TO INTERVIEWER: Read the consent form for in-depth interviews out loud in the participant’s preferred language and give him a copy. Ask him if he has any questions, and answer them. Ask him to sign if he agrees to participate. If the participant agreed to participate, you may start recording here, without recording any names or identifying information.**

1. ***Perceptions, attitudes and preferences related to incentives offered***
   1. I want to start by asking you about the first contact you had with our study team after the initial census visit to your household. Let’s take a minute for you to recall the day our study team member came and told you about the prizes offered to men who come to the Community Health Campaign to participate in HIV testing.
      1. Can you remember that day? Where were you? What were you doing at the time?
      2. Can you tell me, please, what did the study team member tell you about the prize?

- [*for Loss Aversion-based Incentives group*:] How did the prize look to you?
  - 1. And what was your first reaction to learning about this prize?
- And what else were you thinking, about the prize?
- How did it make you feel, to hear about it?
  - 1. Do you remember what else you talked about with the study team member?
- Were there any questions you asked? Were there questions you had, but didn’t ask the team member at that time?
  1. Next I want to ask you about what you did and talked about with important people in your life, about the prize and the HIV testing, after the study team member left. What happened next, that day?
     1. Who did you tell, about the prize you were offered?
- Why did you choose to talk about it with that person?
- And how did they respond, when you told them?
- Were there other people you wanted to talk about it with, but felt you couldn’t? Please tell me more about that.

1. ***Motivations and barriers related to decision to participate in testing***
2. Let me ask you some more about that prize, and about your decision not to come to the Community Health Campaign to participate in HIV testing. How did it affect your decision-making about whether or not to come to the campaign to get the test?
   - 1. Please tell me more about that. *Probe for why the respondent did not come to CHC for HIV testing.*
     2. What other issues did you consider, when you were deciding whether or not to come to the campaign for testing?
     3. Can you tell me, please, about the reasons why you decided not to come to the campaign for testing? *Probe for whether or not the respondent has previously tested for HIV but decided not to test now.*

- Please tell me more about your concerns about getting tested for HIV.
- What do you think would make it easier for you to decide to come to a community health campaign to test for HIV? *Probe for whether respondent would prefer to test for HIV at another location (at a clinic, or at home), and if so, reasons why*.

1. ***Relationship, family and other contextual factors related to incentives and testing***
2. Now I want to ask you about how other men in your community feel about getting tested for HIV, especially your friends. How do you think most men feel about getting tested for HIV?
   - 1. To what extent do you and your friends talk about HIV testing? What kinds of things do your friends say about it?
     2. What are the main motivations for men to get tested for HIV?
     3. Please tell me, what do you think are the main reasons why men do not get tested for HIV?
     4. What other barriers do men face, related to HIV testing?
     5. What about other medical services—how do most men in this community, especially your friends, feel about accessing medical care?
3. I want to ask you more about your main intimate and family relationships, and about how you and your partners have handled discussions around testing for HIV. First, please tell me about your marital status, and about any girlfriends? *Probe for whether respondent has a wife or main partner with whom he resides, and any other sexual partners he sees regularly.*

*Note: Remind respondent about Informed Consent form and human subjects protections. Interviewers should encourage respondents to report on all of their sexual partnerships, including non-marital partners. Probe for whether respondent has a wife or main partner with whom he resides, and any other sexual partners he sees regularly.*

- - 1. [If currently married] Is HIV testing something you and your wife have talked about? Please tell me about that.
- What are the things that make it hard to discuss HIV testing with her?
- What do you think might make it easier?
  - 1. [If respondent has non-marital sexual partners]: Is HIV testing something you have talked about with your girlfriend[s]? Please tell me about that.
- What are the things that make it hard to discuss HIV testing with her?
- What do you think might make it easier?

1. Is there anything else you’d like to share with me, about your experience testing for HIV, or about the prizes offered to men in this study after testing for HIV?

THANK THE RESPONDENT FOR HIS PARTICIPATION, AND COMPLETE REFERRAL PROCESS AS NEEDED.
